# Effect of Ocean Acidification on Bacterial Metabolic Activity and Community Composition in Oligotrophic Oceans, Inferred From Short-Term Bioassays

**DOI:** 10.3389/fmicb.2021.583982

**Published:** 2021-02-26

**Authors:** Caiqin Hu, Xiangfu Li, Maoqiu He, Peng Jiang, Aimin Long, Jie Xu

**Affiliations:** ^1^State Key Laboratory of Tropical Oceanography, South China Sea Institute of Oceanology, Chinese Academy of Sciences, Guangzhou, China; ^2^College of Marine Science, University of Chinese Academy of Sciences, Beijing, China; ^3^Southern Marine Science and Engineering Guangdong Laboratory, Guangzhou, China; ^4^College of Ocean and Earth Sciences, University of Xiamen, Xiamen, China; ^5^Innovation Academy of South China Sea Ecology and Environmental Engineering, Chinese Academy of Sciences, Guangzhou, China

**Keywords:** bacterial metabolic activity, bacterial community composition, microbial carbon cycling, ocean acidification, the northern South China Sea

## Abstract

Increasing anthropogenic CO_2_ emissions in recent decades cause ocean acidification (OA), affecting carbon cycling in oceans by regulating eco-physiological processes of plankton. Heterotrophic bacteria play an important role in carbon cycling in oceans. However, the effect of OA on bacteria in oceans, especially in oligotrophic regions, was not well understood. In our study, the response of bacterial metabolic activity and community composition to OA was assessed by determining bacterial production, respiration, and community composition at the low-*p*CO_2_ (400 ppm) and high-*p*CO_2_ (800 ppm) treatments over the short term at two oligotrophic stations in the northern South China Sea. Bacterial production decreased significantly by 17.1–37.1 % in response to OA, since bacteria with high nucleic acid content preferentially were repressed by OA, which was less abundant under high-*p*CO_2_ treatment. Correspondingly, shifts in bacterial community composition occurred in response to OA, with a high fraction of the small-sized bacteria and high bacterial species diversity in a high-*p*CO_2_ scenario at K11. Bacterial respiration responded to OA differently at both stations, most likely attributed to different physiological responses of the bacterial community to OA. OA mitigated bacterial growth efficiency, and consequently, a larger fraction of DOC entering microbial loops was transferred to CO_2_.

## Introduction

Twenty-three percent of atmospheric CO_2_ released by anthropogenic activities is absorbed by oceans (Le Quere et al., 2018), leading to ocean acidification (OA). Since the industrial revolution, pH of surface seawater has decreased by 0.12 (IPCC, 2014). Atmospheric CO_2_ is predicted to rise to 800–1,000 μatm by the end of the century. pH of surface seawater would fall by 0.4–0.5, and the carbonate system would be affected (Riebesell and Gattuso, 2015). Ocean acidification regulates the eco-physiological response of marine organisms, in terms of their survival, growth, photosynthesis, metabolism, calcification, and nitrogen fixation rate, ultimately likely disturbing marine ecosystems (Pereira, 2020).

Heterotrophic bacteria play an important role in carbon cycling in marine environments, which are responsible for decomposing 75–95% of organic matter (Boyd et al., 1999). Bacterial production (BP) generally accounts for 10–90% of primary production (PP) and even exceeds PP in some oligotrophic regions (Kirchman and Ducklow, 1993). Bacterial respiration (BR) accounts for 50–90% of community respiration (CR) (Biddanda and Cotner, 2002). Ocean acidification is reported to modulate the cell morphology (Yu and Chen, 2019), metabolism (Westwood et al., 2018; Vaque et al., 2019), and community structure (Grossart et al., 2006; James et al., 2019) of marine bacteria, ultimately affecting the carbon flow of the marine microbial loop. Although the effect of ocean acidification on bacterial metabolic activity and community composition has been recognized in the last decades (Riebesell and Gattuso, 2015), contrasting findings are achieved (Weinbauer et al., 2011), likely due to different nutritional conditions or experimental designs.

A recent study in Arctic Ocean has shown that bacterial production increased in high-*p*CO_2_ treatments at low temperatures and low chlorophyll *a* (Chl *a*) concentrations (Vaque et al., 2019). Westwood et al. (2018) report that an increase in BP is attributed to the effect of OA on phytoplankton-derived DOC and the lower grazing pressure in highly productive waters. Xia et al. (2019) suggest that enhanced BP under high *p*CO_2_ is caused by the physiological adjustment of bacterial cells. Instead, in mesopelagic regions, high *p*CO_2_ reduces BP in Hawaiian deep seawater (Coffin et al., 2004). In addition, Huang et al. (2018) find that BP is little affected by OA in algal blooms, because of limited inorganic nutrient supply.

Less attention has been paid to the effect of ocean acidification on bacterial respiration. Elevated bacterial respiration under the high CO_2_ level is observed, accompanied by a rise in bacterial abundance (BA) and production (Fuentes-Lema et al., 2018). In contrast, enhanced *p*CO_2_ causes a distinct decrement of bacterial respiration in an algal bloom but has little effect on bacterial production, leading to high bacterial growth efficiency (BGE) (Huang et al., 2018).

The response of bacterial community composition (BCC) to ocean acidification varies. For example, high *p*CO_2_ has little effect on bacterial community richness or diversity (Roth-Schulze et al., 2018). However, Xia et al. (2019) report that OA reduces bacterial diversity and results in shifts in the bacterial community in a 12-day dark incubation experiment. Bacterial clades of *Pseudoalteromonadaceae* and *Shewanellaceae* increase in the relative abundance in high-*p*CO_2_ treatments, accompanied by a decline in BGE (James et al., 2019).

The response of the bacterial community to OA is regulated by nutrient conditions (Alvarez-Fernandez et al., 2018). Bacteria in oligotrophic conditions should be more sensitive to the change of pH, since they are already suppressed by deficient nutrients (Takeuchi et al., 1997). The influence of OA on the microbial community of the Northwestern Mediterranean Sea is more pronounced when nutrient concentrations are low (Sala et al., 2016). However, high *p*CO_2_ has little effect on plankton metabolism including gross primary production, net community production, and community respiration in the oligo- to meso-trophic Northwestern Mediterranean Sea (Maugendre et al., 2015). In addition, the abundance of heterotrophic prokaryotes is not affected and bacterial production reduces under high *p*CO_2_ in one of mesocosms (Celussi et al., 2017). Hornick et al. (2017) also find no consistent response of bacterial protein production to ocean acidification in low-nutrient conditions. Phylogenetic analysis reveals that ocean acidification decreases the species diversity of under-ice bacteria in the oligotrophic Arctic Ocean, while there is no clear trend (Monier et al., 2014). Hence, the response of the bacterial community to ocean acidification is variable in oligotrophic oceans. Little is known on the mechanism regulating bacterial metabolism under ocean acidification in oceans.

Protistan grazing, as the top-down control of bacterial mortality in oligotrophic ocean regions (Guixa-Boixareu et al., 1996), not only transfers bacterial biomass into higher trophic levels (Mcmanus and Fuhrman, 1988) but also generates growth resources for fueling bacterial activity (Pradeep Ram and Sime-Ngando, 2008). The increasing *p*CO_2_ results in significantly higher prey abundance and grazing rate (Rose et al., 2009). In contrast, a recent study suggests that OA has no measurable effect on micro-/mesozooplankton grazing rates on phytoplankton (Wang et al., 2019). Changes in protistan grazing on bacteria under OA scenario potentially affected the response of bacteria to OA.

In this study, mesocosm experiments were conducted in the oligotrophic northern South China Sea (nSCS), in order to examine the response of bacterial metabolism and community composition to OA in oligotrophic oceans, which helped to improve our understanding of bacterial carbon cycling in the scenario of ocean acidification in marine environments.

## Materials and Methods

### Experimental Setup

**FIGURE 1:**
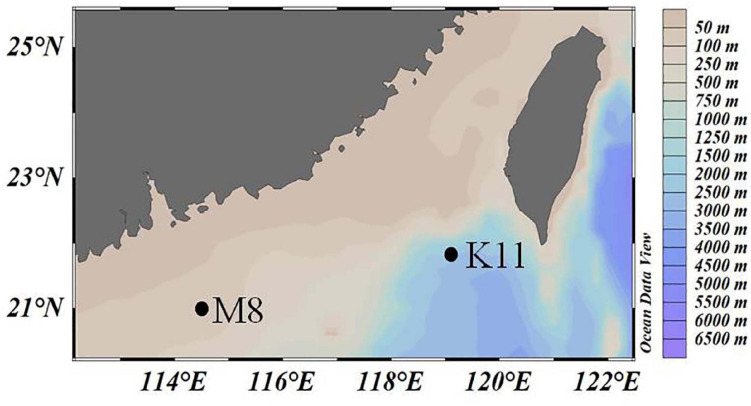
The location of two investigation stations M8 and K11 in the northern South China Sea in June 2019.

Two mesocosm experiments were conducted at oligotrophic stations M8 and K11 in the northern South China Sea in June 2019 (Figure 1), respectively. Temperature and salinity of the ambient seawater were determined with the conductivity–temperature–depth system (CTD, Sea-Bird).

The M8 and K11 stations were located in the continental shelf and slope area in the northern South China Sea, with water depths of 91 and 2,267 m, respectively. Surface temperatures were 30°C at both stations. Surface salinity (32.9) at M8 was slightly lower than that (33.8) at K11. The DIN concentration (0.23 μM) at M8 was slightly higher than that (0.16 μM) at K11. The ambient DOC concentrations at M8 and K11 were 82.5 and 73.5 μM, respectively. The ambient Chl *a* concentration at M8 was 0.13 μg L^–1^, comparable with that (0.11 μg L^–1^) at K11 (Table 1).

**TABLE 1:** The ambient levels of various environmental parameters at stations M8 and K11.

Station	M8	K11
Depth (m)	91	2267
Temperature (°C)	30.2	30.3
Salinity	32.9	33.8
DIN (μM)	0.23	0.16
PO_4_ (μM)	0.01	0.01
SiO_4_ (μM)	1.29	1.30
DOC (μM)	82.5	73.5
Chl *a* (μg L^–1^)	0.13	0.11

Surface seawater was incubated in six 60-L precleaned transparent polyethylene tanks, the inside of which were covered with transparent polyethylene bags (acid cleaned with 10% HCl). Tanks were closed. A tube was used in each tank connecting the atmosphere. There was about 2 L headspace in the tank at the beginning of incubation. Three of six tanks were bubbled with a humidifier and 0.22-μm filtered CO_2_–air mixtures (CO_2_ concentration of 1,000 ppm) that were generated by CO_2_ mixers (CE100-C, Wuhan RUIHUA, China) as the high-*p*CO_2_ treatment (HC), and the other three tanks were pumped with air as the low-*p*CO_2_ treatment (LC). The *p*CO_2_ concentrations of HC and LC maintained at 800 ppm and 400 ppm, respectively (Supplementary Figure 1). Both high CO_2_ and air were pumped into each tank at a flow rate of 0.4–0.6 L min^–1^. Four gas tubes were placed at the top, the top third, the bottom third, and the bottom of each tank, respectively, to evenly disperse the gas into the water column. These gas tubes were connected to plastic air stones (Lee’s, United States) to produce smaller bubbles with the diameter of about 0.2 mm. It was assumed that these small bubbles through the air stones would not disturb the organisms in the mesocosm. The high CO_2_ and air were pumped continuously. *p*CO_2_ was monitored continuously. pH was measured daily using a spectrophotometric method based on the proton exchange behavior of thymol blue (Zhang and Byrne, 1996). Bioassays were incubated in triplicate for 3 days at M8 and 4 days at K11, respectively. The tanks were covered by the neutral-density screens to obtain 50% of the light intensity at the ocean surface. The tanks were placed in a larger transparent tank with running surface seawater to maintain the *in-situ* temperature. Subsamples for bacterial metabolic activity and DNA analysis were collected at the beginning, the second day (Day 2) and the end of incubation.

### Nutrients, Chlorophyll *a*, Dissolved Organic Carbon, and Bioavailable Dissolved Organic Carbon

Seawater for nutrients (NO_3_, NO_2_, NH_4_, PO_4_, and SiO_4_) was filtered through GF/F filters (Whatman, United States) and frozen at −20°C until analyzed by an AA3 auto-analyzer (SEAL, Germany). Dissolved inorganic nitrogen (DIN) concentrations were the sum of NO_3_, NO_2_, and NH_4_ concentrations (Grasshoff et al., 1999).

For Chl *a* samples, 1 L seawater was filtered onto the GF/F filters (Whatman, United States) and then the filters were kept at −20°C. Chl *a* concentrations were measured with a fluorometer (Turner Designs Trilogy, United States) after acetone extraction at 4°C for 24 h (Parsons, 1985).

Water samples for Dissolved organic carbon (DOC) were filtered through pre-combusted (450°C for 4 h) GF/F filters (Whatman, United States) and stored in pre-combusted glass vials at −20°C. DOC concentrations were analyzed with an auto-analyzer (Shimadzu TOC-VCPH, Japan) (Alvarez-Salgado and Miller, 1998).

Bioavailable dissolved organic carbon at the end of the incubation in mesocosm experiments was determined by the declined DOC concentrations over the 30-day incubation at room temperature.

### Bacterial Abundance, Production, and Respiration

For bacterial abundance, triplicate seawater samples from each tank were fixed with glutaraldehyde (final concentration 0.5% v/v), flash-frozen in liquid nitrogen, and then stored at −80°C until analyzed. Bacterial abundance was counted by flow cytometry (Becton-Dickinson Accuri^TM^ C6) after being stained with SYBR Green-I according to Marie et al. (1997), with a scatter plot of SSC vs. FL1 and a scatter plot of FL1 vs. FL3. The bacterial community was divided into low nucleic acid (LNA) and high nucleic acid (HNA) groups (Gasol et al., 1999). The relative cell size of bacteria was estimated by the forward scatter (FSC) (Jiao and Yang, 1999).

Bacterial production was measured by the ^3^H-leucine tracer method (Knap et al., 1996). Triplicate seawater samples from each tank were added with ^3^H-leucine (final concentration 27 nM, 54.1 Ci mmol^–1^) and then incubated in the dark for 3 h and terminated by adding TCA reagent (5% v/v final concentration). The blank was obtained by adding TCA reagent into seawater before the incubation. BP was determined with a PerkElmer liquid flash counter (Tri-Carb 2810, United States), after the samples were centrifuged following the procedures described by Kirchman (2001). Bacterial production was calculated with the leucine-to-carbon empirical conversion factors of 1.5 kg C⋅mol leucine^–1^ (Kirchman, 1993). The cell-specific bacterial production (sBP) was calculated by dividing BP by BA.

Bacterial respiration and plankton community respiration were determined according to the electron transport system (ETS) activity–INT reduction method (Martinez-Garcia et al., 2009). Triplicate seawater samples from each tank were incubated in the dark for 6 h after adding the INT solution [tetrazolium salt 2-para (iodo-phenyl)-3(nitrophenyl)-5(phenyl) tetrazolium chloride, final concentration 0.2 mM]. Subsequently, seawater samples were fixed with formaldehyde and filtrated onto 1.0 and 0.2-μm polycarbonate filters (Whatman, United States), sequentially. The filters were stored at −20°C. The INT reduced in each size fraction was extracted with propanol and the absorbance at 485 nm determined using a spectrophotometer (PGENERAL, TU-1810). Bacterial respiration (BR_*INT–F*_) was derived from the INT reduction in the 0.2-μm fraction. Plankton community respiration (CR_*INT–F*_) was regarded as the sum of the INT reduction in the 1.0-μm and 0.2-μm fractions. The respiration rate in the >1.0-μm fractions resulted mainly from eukaryotes and particle-attached prokaryotes, while the main respiring organisms in the 0.2–1.0-μm fractions are heterotrophic bacteria (Martinez-Garcia et al., 2009). The oxygen consumption to an INT reduction ratio (R/ETS) of 12.4 was used in this study (Supplementary Figure 2), which was derived from the plankton community respiration measured by INT reduction and Winkler-based O_2_ consumption (Oudot et al., 1988) methods from 103 discrete samples in the South China Sea (Hu et al., in preparation). Bacterial contribution to plankton community respiration (BR%) was estimated by dividing CR by BR. The cell-specific bacterial respiration (sBR) was calculated by dividing BR by BA.

Bacterial carbon demand (BCD) was regarded as the sum of BP and BR. Over the incubation time, BGE was calculated from time-integrated BP and BR (IBP and IBR), following the equation: $\text{BGE}=\frac{\text{IBP}}{\text{IBP}+\text{IBR}}$.

### Bacterial Community Composition

Seawater samples (1 L) were filtered onto 0.2-μm polycarbonate filters (Millipore, United States), which were immediately placed into liquid nitrogen and then stored at −80°C. Triplicate samples for DNA were taken on Day 2 and at the end of the incubation, while no replicate samples for DNA were collected at the beginning of the incubation. V3 and V4 fragments of 16S rRNA genes were amplified using primer set 341F (CCTAYGGGRBGCASCAG) and primer 806R (GGACTACNNGGGTATCTAAT) (Behrendt et al., 2012) with a 12-bp barcode. PCR reactions, containing 25 μl 2 × Premix Taq (Takara Biotechnology, Dalian Co., Ltd., China), 1 μl each primer (10 mM), and 3 μl DNA (20 ng μl^–1^) template in a volume of 50 μl, were amplified by thermocycling: 5 min at 94°C for initialization, 30 cycles of 30 s denaturation at 94°C, 30 s annealing at 52°C, and 30 s extension at 72°C, followed by 10 min final elongation at 72°C. The PCR instrument was BioRad S1000 (Bio-Rad Laboratory, CA, United States). PCR products were mixed in equidensity ratios according to the GeneTools Analysis Software (Version 4.03.05.0, SynGene). Then, mixture PCR products were purified with E.Z.N.A. Gel Extraction Kit (Omega, United States). Sequencing libraries were generated using NEBNext Ultra^TM^ II DNA Library Prep Kit for Illumina (New England Biolabs, MA, United States). The library was sequenced on an Illumina Nova6000 platform and 250-bp paired-end reads were generated.

Fastp^1^ (version 0.14.1) was used to analyze the gene data. For each representative sequence, the Silva database was used to annotate taxonomic information by usearch-sintax (set the confidence threshold to default to more than 0.8). The operational taxonomic units (OTUs) which were annotated as chloroplasts or mitochondria were removed. The Silva database was also applied for classifying the operational taxonomic units. Bacterial species with the relative abundance being more than 0.5% of total OTUs were analyzed. The Chao1 richness and Shannon diversity indices were calculated at a level of 97% similarity. Bacterial diversity was converted at the base of natural logarithm e. The relative abundance of bacterial species for each sample was calculated at the phylum and family levels. The relative abundance of each phylum and the most abundant families (relative abundance > 0.5%) in replicate samples of community composition was averaged. The statistically significant difference in the relative abundance of individual species between treatments at each sampling time point was determined in pairs by the t-test analysis at the *p* < 0.05 level with SPSS software. The significant difference in the averaged relative abundance of individual species between treatments and different sampling time points were determined by the F-test of two-way ANOVA analysis at the *p* < 0.05 level with SPSS software. The network analysis was performed to determine the relationship of the bacterial metabolic parameters (BA, BP, BR, CR, BR%, BCD, and BGE) and the abundance (the reads of OTUs) of the most abundant species (at family level, the relative abundance more than 0.5%) between high-*p*CO_2_ treatment and low-*p*CO_2_ treatment at each sampling time point (Day 2 and Day 3 for M8, Day 2 and Day 4 for K11). The Pearson correlation between bacterial metabolic parameters and the abundance of species was performed in pairs with R package Cor. The network of each station was based on the pairwise Spearman correlation. Only a strong correlation (*R* > 0.7 and *R* < −0.7, *p* < 0.05) was visualized through network analysis using Cytoscape software (Shannon et al., 2003). Data on bacterial community composition on Day 0 were not included in the network analysis. All the sequences obtained from this study have been deposited in the National Center for Biotechnology Information Sequence Read Archive under accession number: SAMN16203441–SAMN16203465.

### Grazing Rate of Protists on Bacteria

Grazing rates on bacteria by protists of the total plankton community were determined by the fluorescently labeled bacteria (FLB) disappearance method (Vazquez-Dominguez et al., 1999). The cultured *Brevundimonas diminuta* which was heat-killed and stained with [5-(4,6-dichlorotriazin-2yl)amino]-fluorescein was added into three 2-L polycarbonate bottles, two of which were filled with 1 L seawater (filtered through a 200-μm mesh to remove large zooplankton), and the other bottle was filled with 1 L bacteria-free seawater (filtered through 0.2-μm filters) as the control. Bottles were incubated in duplicate in the dark for 24 h at *in-situ* temperature. Subsamples for the abundance of FLB were taken at the beginning (F_0_) and at the end of incubations (F_*t*_). The abundance of FLB was determined with epifluorescence microscopy (Nikon Ci-S, 1,000 X magnification) under the blue light. The specific grazing rates (g) during incubation time (t) were calculated according to the equation of Boras et al. (2010):

$$g=-\left(\frac{1}{t}\right)\ln\left(\frac{F_{t}}{F_{0}}\right).$$

### Statistical Analyses

The statistical significance of difference between treatments at each sampling time point of each station was determined in pairs by the t-test analysis performed with SPSS software at the *p* < 0.05 level. The Benjamini–Holmberg correction was employed to correct the *p*-values of the t-test and two-way ANOVA analysis using the p.adjust function of states package in R software. Triplicate samples were averaged for environmental variables (pH, Chl *a*, DOC, and BDOC) and for bacterial metabolic variables (bacterial abundance, production, respiration, and community composition) in each tank, while duplicate samples were averaged for the protistan grazing rate in each tank. As one tank in high-*p*CO_2_ treatment at K11 was fallen over on Day 4, the bacterial abundance, production, respiration, and community composition were averaged from the remaining duplicate samples in HC treatment (*n* = 2). For other treatments, variables were calculated from triplicate samples (*n* = 3). The standard deviations (± SD) were calculated for these variables.

## Results

### Environmental Conditions

**FIGURE 2:**
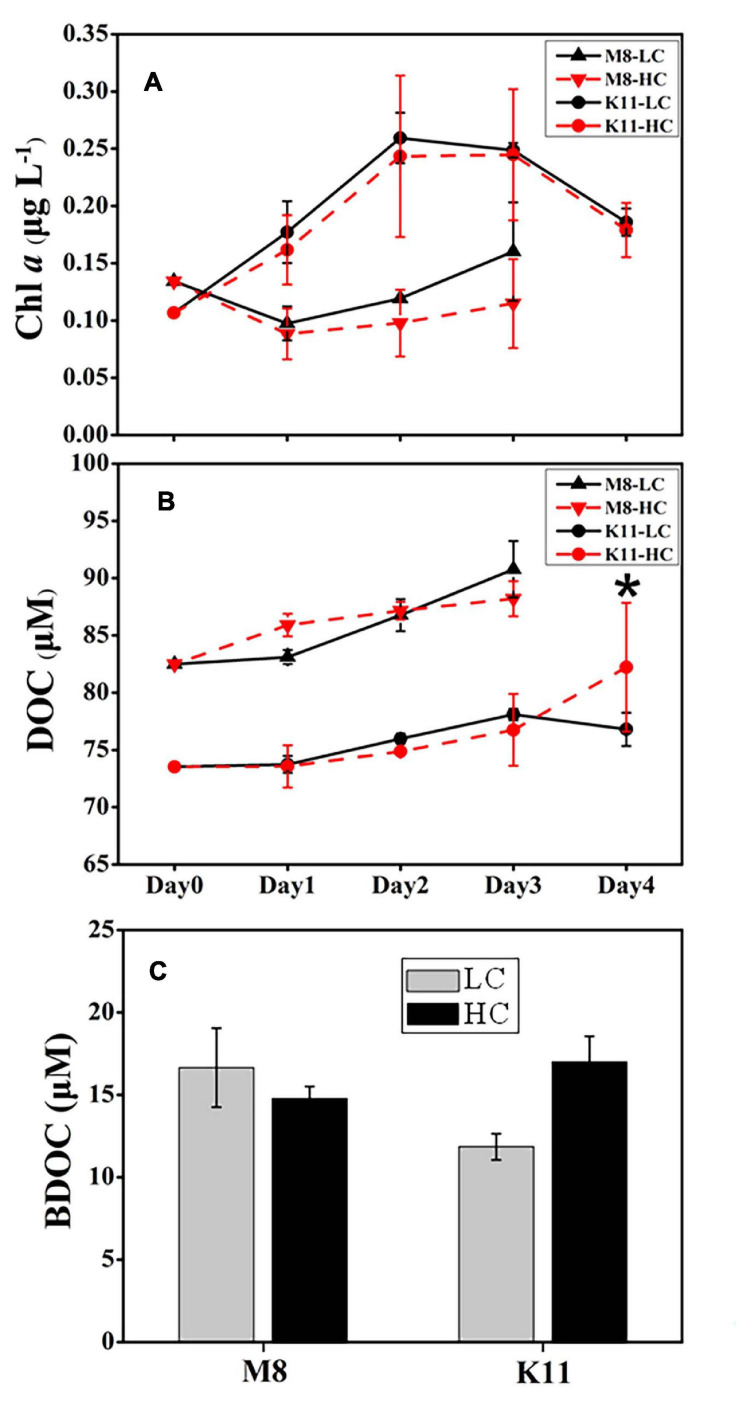
Changes in concentrations of chlorophyll *a* **(A)** and dissolved organic carbon **(B)** during the incubation and bioavailable dissolved organic carbon **(C)** at the end of the incubation at Stations M8 and K11. LC: control treatments. HC: high-*p*CO_2_ treatments. Asterisk denoted the significant differences (*p* < 0.05) between LC and HC. The error bars represented ± SD. For samples of HC at K11 on Day 4, *n* = 2. For other treatments, *n* = 3.

The pH remained stable (8.02 ± 0.03 in LC and 7.84 ± 0.02 in HC) throughout the experimental period (Supplementary Figure 1). During incubation, the Chl *a* concentration at both stations displayed an opposite pattern (Figure 2A). The Chl *a* concentration at M8 dropped to less than 0.10 μg L^–1^ on Day 1 and then increased slowly. In contrast, the Chl *a* concentration at K11 increased to 0.26 μg L^–1^ in the first 2 days of incubation and then declined. The Chl *a* concentration at K11 was higher than that at M8 throughout the incubation. There was no significant difference in Chl *a* concentration between HC and LC. The DOC concentration at both stations increased during the incubation. At the end of the incubation, there was no significant difference in BDOC between LC and HC at two stations. However, BDOC (16.7 ± 2.40 μM) in LC was slightly higher than (14.8 ± 0.73 μM) in HC at M8 on Day 3, while BDOC (11.9 ± 0.80 μM) in LC was slightly lower than (17.0 ± 1.54 μM) in HC at K11 on Day 4 (Figure 2C).

### Bacterial Metabolic Activities

**FIGURE 3:**
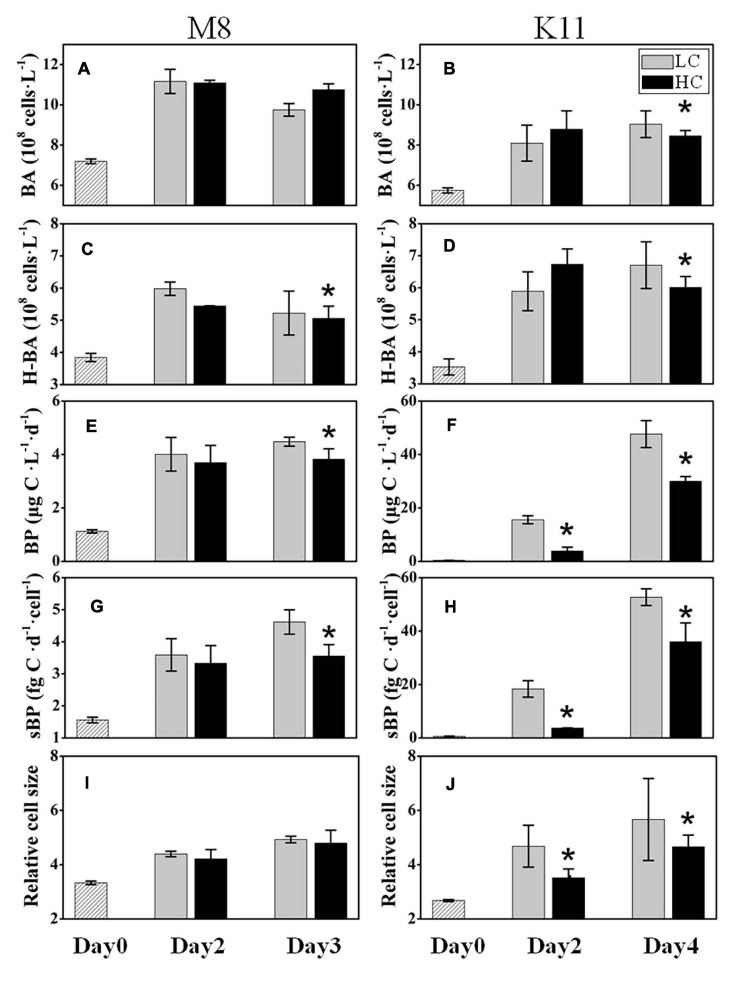
Bacterial abundance (BA) **(A,B)**, high nucleic acid bacterial abundance (H-BA) **(C,D)**, bacterial production (BP) **(E,F)**, cell-specific bacterial production (sBP) **(G,H)**, and bacterial relative cell size **(I,J)** at M8 and K11 in mesocosm experiments. LC, control treatments; HC, high-*p*CO_2_ treatments. Asterisk denoted the significant (*p* < 0.05) difference between LC and HC. The error bars represented ±SD. For samples of HC at K11 on Day 4, *n* = 2. For other treatments, *n* = 3.

The initial BA at stations M8 and K11 were 7.19 ± 0.12 × 10^8^ and 5.75 ± 0.13 × 10^8^ cells L^–1^, respectively. BA in LC increased 1.36 times at M8 and 1.57 times at K11 at the end of the incubation, respectively (Figures 3A,B). At the end of incubation, BA (8.45 ± 0.27 × 10^8^ cells L^–1^) in HC was significantly lower than (9.04 ± 0.67 × 10^8^ cells L^–1^) in LC at K11. The abundance of HNA bacteria (H-BA) at M8 was lower in HC (5.06 ± 0.38 × 10^8^ cells L^–1^) than in LC (5.22 ± 0.68 × 10^8^ cells L^–1^) on Day 3. At the end of incubation, the H-BA at K11 in HC was 6.01 ± 0.34 × 10^8^ cells L^–1^, significantly lower than that (6.71 ± 0.64 × 10^8^ cells L^–1^) in LC (Figures 3C,D).

The initial BP at M8 was 1.12 ± 0.06 μg C L^–1^ d^–1^ and increased fourfold in LC at the end of the incubation. BP at K11 was 0.35 ± 0.02 μg C L^–1^ d^–1^ at the beginning of the incubation and increased significantly to 47.7 ± 5.05 μg C L^–1^ in LC after 4 days of incubation. Nevertheless, at the end of the incubation, BP (3.82 ± 0.39 μg C L^–1^) in HC at M8 was significantly lower than (4.48 ± 0.17 μg C L^–1^) in LC (Figure 3E). At K11, BP (3.90 ± 1.38 μg C L^–1^) in HC was significantly lower than (15.6 ± 1.49 μg C L^–1^) in LC on Day 2 (Figure 3F). sBP exhibited significantly lower (3.56 ± 0.36 fg C d^–1^ cell^–1^ at M8 on Day 3 and 3.70 ± 0.02 fg C d^–1^ cell^–1^ at K11 on Day 2) in HC than (4.62 ± 0.38 fg C d^–1^ cell^–1^ at M8 and 18.3 ± 3.12 fg C d^–1^ cell^–1^ at K11) in LC at both stations (Figures 3G,H). The relative cell size of bacteria was lower in HC than in LC at K11, but not at M8 (Figures 3I,J).

**FIGURE 4:**
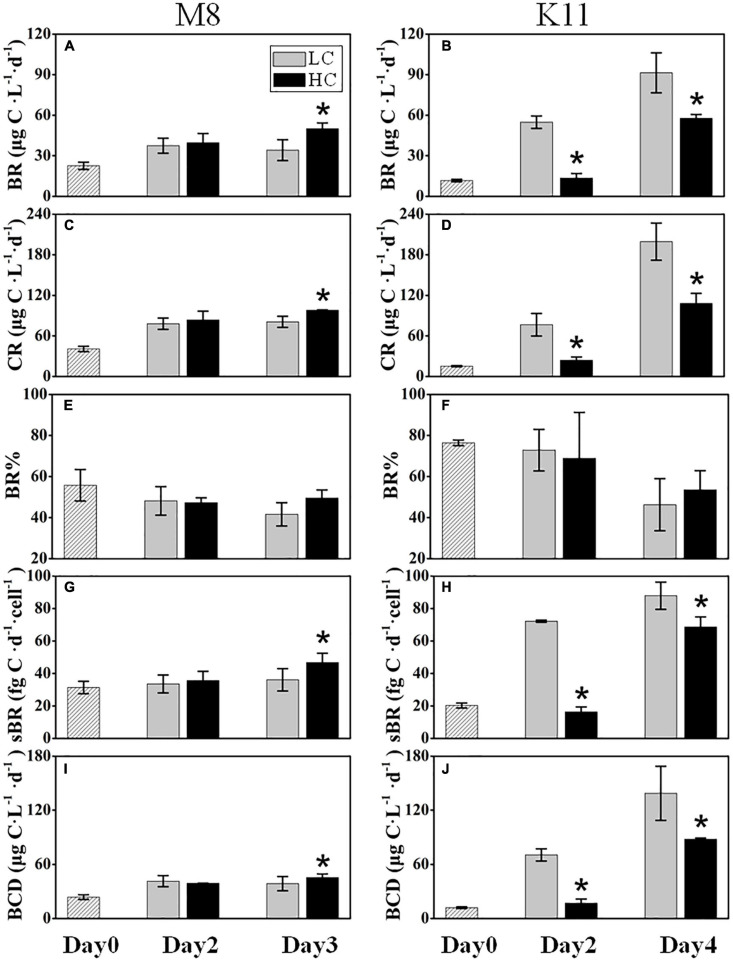
Bacterial respiration (BR) **(A,B)**, plankton community respiration (CR) **(C,D)**, bacterial contribution to total plankton community respiration (BR%) **(E,F)**, cell-specific bacterial respiration (sBR) **(G,H)**, and bacterial carbon demand (BCD) **(I,J)** at M8 and K11 in mesocosm experiments. LC, control treatments; HC, high-*p*CO_2_ treatments. Asterisk denoted the significant (*p* < 0.05) difference between LC and HC. The error bars represented ±SD. For samples of HC at K11 on Day 4, *n* = 2. For other treatments, *n* = 3.

BR at M8 and K11 at the beginning of the incubation were 22.6 ± 2.71 μg C L^–1^ d^–1^ and 11.7 ± 0.88 μg C L^–1^ d^–1^, respectively. BR in HC (50.1 ± 4.15 μg C L^–1^ d^–1^) at M8 was about 1.5 times higher than (34.1 ± 7.71 μg C L^–1^ d^–1^) in LC on Day 3, while at K11, BR was lower in HC (13.5 ± 3.39 μg C L^–1^ d^–1^) than (54.8 ± 4.61 μg C L^–1^ d^–1^) in LC on Day 2 (Figures 4A,B). Similar to BR, after the incubation, CR displayed a higher value of 98.0 ± 0.59 μg C L^–1^ d^–1^ in HC than (80.8 ± 8.30 μg C L^–1^ d^–1^) in LC at M8 on Day 3, and lower CR value of 24.0 ± 4.93 μg C L^–1^ d^–1^ was detected in HC than (76.6 ± 16.7 μg C L^–1^ d^–1^) in LC at K11 on Day 2 (Figures 4C,D). There was no significant difference in BR% between HC and LC at both stations (Figures 4E,F). On Day 3, the sBR was 36.2 ± 6.88 fg C d^–1^ cell^–1^ in LC, lower than (46.8 ± 5.65 fg C d^–1^ cell^–1^) in HC at M8. sBR was 72.2 ± 0.64 fg C d^–1^ cell^–1^ in LC, higher than (16.4 ± 3.00 fg C d^–1^ cell^–1^) in HC at K11 on Day 2 (Figures 4G,H).

**FIGURE 5:**
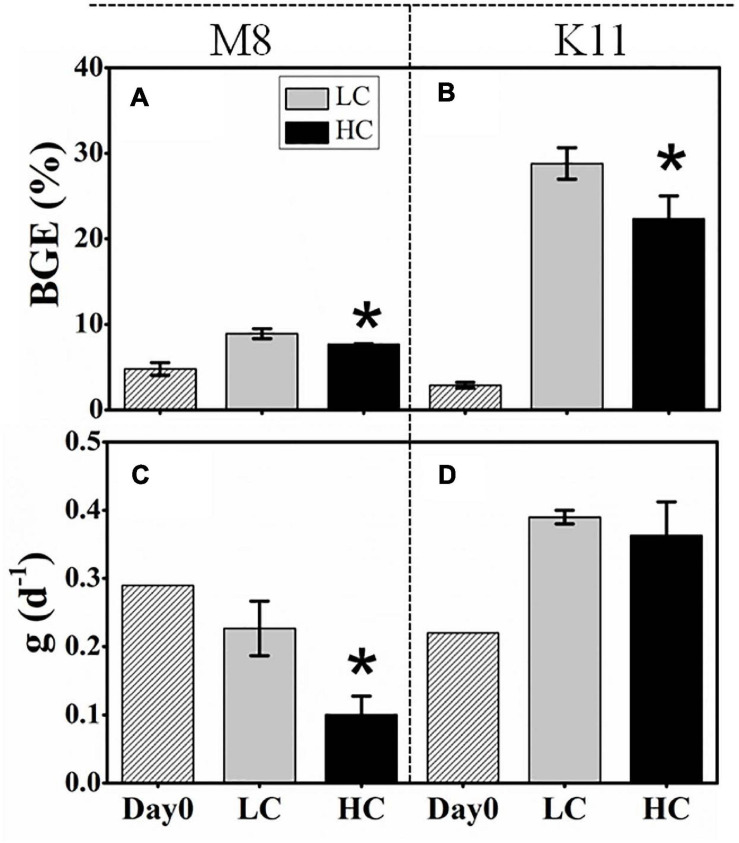
Bacterial growth efficiency (BGE) **(A,B)** at the beginning and calculated from time-integrated BP and BR (IBP and IBR) over the incubation and the specific grazing rate (g) **(C,D)** of protists at the beginning and the end of incubation between treatments at M8 and K11. LC, control treatments; HC, high *p*CO_2_ treatments. Asterisk denoted the significant (*p* < 0.05) difference between LC and HC. The error bars represented ± SD. For samples of HC at K11 on Day 4, *n* = 2. For other treatments, *n* = 3.

BCD (45.4 ± 3.97 μg C L^–1^ d^–1^) in HC at M8 on Day 3 was higher than (38.7 ± 7.90 μg C L^–1^ d^–1^) in LC, while at K11, BCD declined sharply from 70.5 ± 6.71 μg C L^–1^ d^–1^ in LC to 17.0 ± 4.55 μg C L^–1^ d^–1^ in HC on Day 2 (Figures 4I,J). The initial BGE at M8 and K11 were 4.80 ± 0.70% and 2.90 ± 0.30%, respectively. BGE (8.91 ± 0.58% at M8 and 28.8 ± 1.85% at K11) in LC was significantly higher than (7.71 ± 0.02% at M8 and 22.3 ± 2.70% at K11) in HC at M8 and K11 (Figures 5A,B).

### Bacterial Community Composition

**FIGURE 6:**
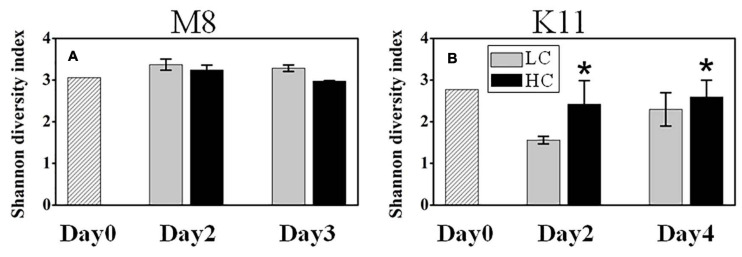
Bacterial diversity at M8 **(A)** and at K11 **(B)** in mesocosm experiments. LC: control treatments. HC: high *p*CO_2_ treatments. Asterisk denoted the significant (*p* < 0.05) difference between LC and HC. The error bars represented ± SD. For samples of HC at K11 on Day 4, *n* = 2. For other treatments, *n* = 3.

**FIGURE 7:**
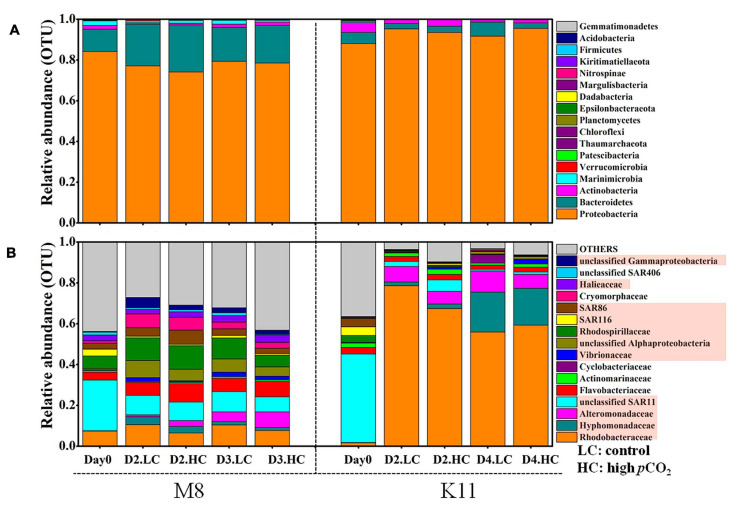
Bacterial community composition at the phylum level **(A)** and the most abundant bacterial families (average relative abundance > 0.5%) **(B)** between treatments at M8 and K11. D2.HC: high *p*CO_2_ treatment on Day 2. Proteobacteria were displayed in red boxes. For samples of HC at K11 on Day 4, *n* = 2. For other treatments, *n* = 3.

1708,116 16S rRNA gene sequences were obtained from the sequencing of 25 samples (one HC tank of K11 was fallen over on Day 4) and were clustered into 731 OTUs. The rarefaction curves reached plateau (Supplementary Figure 3). The bacterial species richness (Supplementary Figure 4) and diversity (Figure 6A) did not differ between HC and LC at M8. However, the high *p*CO_2_ led to an increment in species diversity (1.56 ± 0.09 in LC and 2.42 ± 0.57 in HC) at K11 after 2 days of incubation (Figure 6B). The *in-situ* bacterial community compositions of M8 and K11 were distinct and changed observably after the incubation, among which the shifts of K11 were more noticeable. There was no significant difference (*p* > 0.05, two-way ANOVA) in averaged relative abundance of bacterial species between HC and LC (Figure 7).

At the phylum level, Proteobacteria dominated (the relative abundance of 84.1%) in the relative abundance at M8, followed by Bacteroidetes (11.0%) and Marinimicrobia (2.18%) (Figure 7A). During the incubation, Proteobacteria decreased in relative abundance, while Bacteroidetes increased at M8. The averaged relative abundance of Bacteroidetes at M8 was higher after the incubation (*F* = 228, *p* < 0.05, two-way ANOVA). Similarly, Proteobacteria (88.1%) dominated, followed by Bacteroidetes (5.41%) and Actinobacteria (4.93%) at K11. Contrary to M8, the relative abundance of Proteobacteria increased, while Actinobacteria declined during the incubation at K11. At the phylum level, OA had little effect on bacterial community composition, except for K11 on Day 4, when Bacteroidetes in HC (relative abundance of 2.67 ± 1.75%) were significantly lower than in LC (6.91 ± 1.23%).

At the family level, *SAR11* was dominant *in situ* at M8, with the relative abundance of 24.8%, followed by *Rhodobacteraceae* (7.40%) and *Rhodospirillaceae* (6.07%). After the incubation, the relative abundance of *Rhodobacteraceae*, *Rhodospirillaceae*, *Flavobacteriaceae*, and *Cryomorphaceae* increased, while *SAR11* and *SAR116* reduced (Figure 7B and Table 2). OA had effects on bacterial species at the family level of M8. *Rhodobacteraceae*, *Rhodospirillaceae*, and *SAR116* exhibited a negative response to acidification, the relative abundance of which dropped from 10.4 ± 3.67%, 10.3 ± 1.32%, and 1.24 ± 0.06% in LC to 7.66 ± 0.63%, 5.84 ± 1.16%, and 0.76 ± 0.20% in HC on Day 3, respectively. In contrast, *Alteromonadaceae* was positively stimulated by OA, with higher relative abundance in HC (2.81 ± 0.68% on Day 2 and 4.63 ± 2.15% on Day 3) than in LC (1.00 ± 0.14% on Day 2 and 1.67 ± 0.21% on Day 3). *SAR86* was higher in relative abundance in HC (6.90 ± 2.05%) than in LC (4.09 ± 0.05%) on Day 2 but did not differ between LC and HC on Day 3. *Flavobacteriaceae* belonging to Bacteroidetes was stable in relative abundance about 6.46 ± 1.34% in LC during incubation but varied in HC (11.5 ± 0.73% on Day 2 and 3.40 ± 0.88% on Day 3).

**TABLE 2:** The average relative abundance (%) (± SD) of most abundant (relative abundance more than 0.5%) family-level clades for bacterial communities during the incubation at two stations.

Family	M8					K11				
	D0	D2.LC	D2.HC	D3.LC	D3.HC	D0	D2.LC	D2.HC	D4.LC	D4.HC
Rhodobacteraceae	7.40	10.5 ± 3.28	6.54 ± 0.49*	10.4 ± 3.67	7.66 ± 0.63*	1.59	78.6 ± 4.16	67.4 ± 17.5*	55.9 ± 9.63	59.3 ± 8.86
Hyphomonadaceae	0.24	3.91 ± 0.94	3.22 ± 1.16	1.61 ± 0.31	1.44 ± 0.40	0.14	1.78 ± 1.08	2.17 ± 1.14	19.5 ± 7.38	18.0 ± 8.44
Alteromonadaceae	0.04	1.00 ± 0.14	2.81 ± 0.68*	1.67 ± 0.21	4.63 ± 2.15*	0.09	9.23 ± 2.80	4.18 ± 0.54*	13.6 ± 4.82	6.76 ± 0.59*
Unclassified SAR11	24.8	9.46 ± 0.37	9.09 ± 2.30	9.97 ± 1.47	7.36 ± 2.36	43.3	2.38 ± 0.67	5.58 ± 2.60*	0.89 ± 0.32	1.17 ± 0.42
Flavobacteriaceae	3.90	6.45 ± 1.45	11.5 ± 0.73*	6.47 ± 0.77	3.40 ± 0.88*	3.28	2.53 ± 0.91	2.72 ± 1.12	1.93 ± 0.78	2.40 ± 1.11
Actinomarinaceae	0.77	0.43 ± 0.18	0.56 ± 0.25	0.77 ± 0.23	0.76 ± 0.88	2.14	1.68 ± 0.76	2.62 ± 1.72	0.69 ± 0.80	1.52 ± 0.55
Vibrionaceae	0.32	1.87 ± 0.75	0.91 ± 0.30*	2.22 ± 0.32	1.83 ± 0.34	0.25	0.31 ± 0.15	0.96 ± 0.08	0.29 ± 0.25	3.70 ± 1.63*
Unclassified alphaproteobacteria	0.72	8.36 ± 1.35	5.57 ± 0.71*	6.38 ± 1.08	4.54 ± 0.83	0.06	0.27 ± 0.11	0.42 ± 0.09*	1.57 ± 0.64	1.18 ± 0.32
Rhodospirillaceae	6.07	11.1 ± 2.29	11.5 ± 0.71	10.3 ± 1.32	5.84 ± 1.23*	3.32	0.45 ± 0.13	0.63 ± 0.13	0.27 ± 0.13	0.34 ± 0.14
SAR116	3.30	0.82 ± 0.12	0.75 ± 0.11	1.24 ± 0.06	0.76 ± 0.02*	4.34	0.28 ± 0.19	0.66 ± 0.14*	0.08 ± 0.03	0.18 ± 0.11
SAR 86	2.93	4.09 ± 0.05	6.90 ± 2.05*	3.24 ± 0.69	2.63 ± 0.20	4.07	0.18 ± 0.04	0.52 ± 0.23*	0.10 ± 0.03	0.13 ± 0.01
Cryomorphaceae	1.31	6.80 ± 2.29	6.27 ± 0.41	3.43 ± 1.75	2.87 ± 0.55	0.08	0.02 ± 0.01	0.02 ± 0.01	0.02 ± 0.01	0.02 ± 0.00
Halieaceae	2.70	2.21 ± 0.28	2.65 ± 0.63	3.19 ± 0.81	3.35 ± 1.03	0.35	0.05 ± 0.01	0.33 ± 0.13	0.05 ± 0.01	0.26 ± 0.11
Unclassified SAR406	1.36	0.70 ± 0.39	0.98 ± 0.14	1.37 ± 0.66	0.50 ± 0.37*	0.43	0.05 ± 0.02	0.06 ± 0.02	0.03 ± 0.02	0.04 ± 0.02
Unclassified gammaproteobacteria	0.41	5.08 ± 0.97	2.32 ± 0.31*	2.43 ± 0.13	2.10 ± 1.05	0.08	0.03 ± 0.00	0.04 ± 0.01	0.06 ± 0.02	0.04 ± 0.02
Others	43.8	27.2 ± 3.74	28.5 ± 1.40	35.3 ± 2.50	50.3 ± 5.48*	36.5	2.09 ± 1.45	11.7 ± 5.18*	4.98 ± 1.71	4.93 ± 0.64

* indicates the significant (*p* < 0.05) difference between control (LC) and high-*p*CO_2_ treatments (HC). D2.HC: high-*p*CO_2_ treatment on Day 2. For samples of HC at K11 on Day 4, *n* = 2. For other treatments, *n* = 3.

At K11, *SAR11* dominated initially, with the relative abundance of 43.3%, followed by *SAR116* (4.34%) and *SAR86* (4.07%). *Rhodobacteraceae* (78.6 ± 4.16%) and *Alteromonadaceae* (9.23 ± 2.80%) became dominant on Day 2. *Hyphomonadaceae* (19.5 ± 7.38%) increased dramatically and became the second most abundant species on Day 4 (*F* = 197, *p* < 0.05, two-way ANOVA). The averaged relative abundance of *SAR116* (*F* = 252, *p* < 0.05, two-way ANOVA) and *SAR86* (*F* = 233, *p* < 0.05, two-way ANOVA) increased from Day 0 to Day 4. OA had negative impacts on the dominant species *Rhodobacteraceae* on Day 2 of K11, with lower relative abundance of 67.4 ± 17.5% in HC than that (78.6 ± 4.16%) in LC. OA also inhibited the growth of *Alteromonadaceae*, with the relative abundance being lower in HC (4.18 ± 0.54%) than in LC (9.23 ± 2.80%) on Day 2. On the contrary, *SAR11*, *SAR116*, and *SAR86* increased their relative abundance under high *p*CO_2_ on Day 2, from 2.38 ± 0.67%, 0.28 ± 0.19% and 0.18 ± 0.04% in LC to 5.58 ± 2.60%, 0.66 ± 0.14% and 0.52 ± 0.23% in HC, respectively.

**FIGURE 8:**
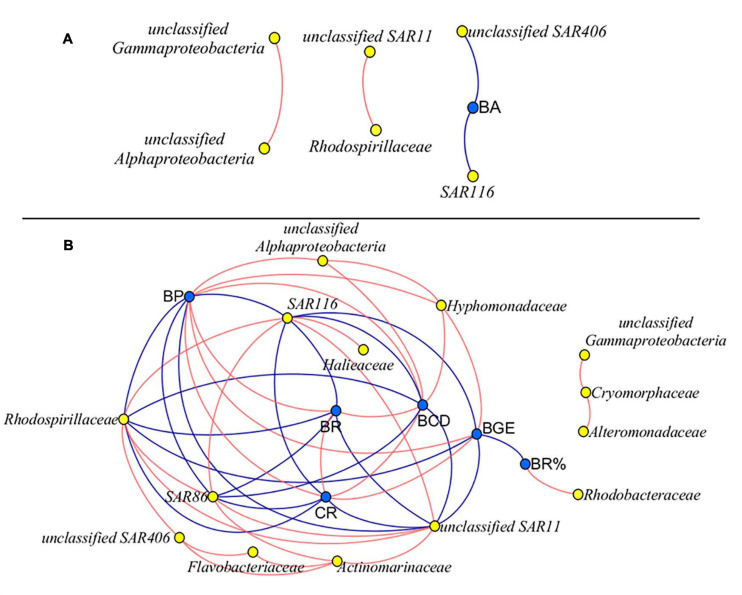
Network analysis between bacterial metabolic activity and community composition at M8 **(A)** and K11 **(B)**, respectively. Data on bacterial species on Day 0 were not included. Blue nodes: bacterial metabolic parameters, yellow nodes: bacterial species. Red lines: positive correlation, blue lines: negative correlation. *R* > 0.7 and *R* < −0.7, *p* < 0.05. For samples of HC at K11 on Day 4, *n* = 2. For other treatments, *n* = 3.

The network analysis revealed that there was no correlation between bacterial metabolism and species, except the negatively significantly correlation between the abundance of *SAR116*, *SAR406*, and BA at M8 (Figure 8A). At K11, there was a negative correlation between BP, BR, CR, BCD, and BGE and the abundance of *SAR11*, *SAR116*, and *Rhodospirillaceae*. BP, BR, CR, and BCD were negatively correlated with the abundance of *SAR86*. BP, BCD, and BGE were positively correlated with the abundance of *Hyphomonadaceae*. The positive relationship between *Rhodobacteraceae* and BR% was found (Figure 8B).

### Grazing Rate

At the beginning of incubation, the grazing rate of protists at M8 was 0.29 d^–1^, higher than that (0.22 d^–1^) at K11. After the incubation, the grazing rate reduced at M8, but rose at K11. The grazing rate in HC (0.10 ± 0.03 d^–1^) was significantly lower than that (0.23 ± 0.04 d^–1^) in LC at M8 (Figures 5C,D).

## Discussion

### Effect of Ocean Acidification on Bacterial Metabolism

During the incubation, BP and sBP increased significantly in LC and HC at both stations, which were associated with an increase in BA, especially H-BA (Figure 3). At K11, BP increased 10-folds during the incubation, accompanied by shifts in bacterial community composition (Figure 7). In contrast, BP at M8 only elevated about threefold after the incubation. This phenomenon of “Bottle Effect” is typically observed in mesocosm experiments, which manifests that the natural bacterial community is rapidly replaced by opportunistic bacteria (Baltar et al., 2012) and bacterial activities enhance considerably (Calvo-Diaz et al., 2011). The difference in BP between M8 and K11 after the incubation was most possibly related to DOC availability and shifts in bacterial community composition. A decrease in Chl *a* concentration (Figure 2A) was indicative of the decay of phytoplankton at M8. Our suggestion was supported by a high relative abundance of *Flavobacteriaceae* at M8, which are often dominant in the senescent phase of algal blooms (Pinhassi et al., 2004; Teira et al., 2008). In contrast, an increase in Chl *a* concentration during the incubation at K11 reflected that phytoplankton was in the growing phase. Phytoplankton-derived DOC in the growing stage is a highly bioavailable and high-molecular-weight (HMW) DOC, such as polymer carbohydrates, improving BP (Hama and Yanagi, 2001; Gobler and Sanudo-Wilhelmy, 2003). In contrast, chitin-containing compounds prevail in DOC at the end of algal blooms (Forest et al., 2008), which was less bioavailable than phytoplankton-derived protein-containing compounds in the growing phase (Shi et al., 2019). Although Chl *a* at K11 increased during the incubation, the DOC concentration at K11 was lower than at M8 throughout the incubation (Figure 2). This was more likely attributed to a higher carbon consumption rate of the bacterial community at K11. The difference in sBP between M8 and K11 also likely resulted from the discrepancy in bacterial community composition, as sBP is related to shifts in bacterial community composition (Xu et al., 2018). *Rhodobacteraceae* were the dominant species at K11 after the incubation, which is a typical high nucleic acid bacteria (Vila-Costa et al., 2012). Lebaron et al. (2002) and Servais et al. (2003) suggest that most of HNA bacteria are highly active in incorporating the substrates.

H-BA decreased due to OA at M8, suggesting that bacteria with low nucleic acid and low activity were numerically dominant in high-*p*CO_2_ treatment (Figures 3A,C). Ocean acidification led to a reduction in BP and sBP at both stations, due to the decreased abundance of bacteria with high nucleic acid (H-BA) in the high-*p*CO_2_ treatment. Changes in H-BA are closely related to BP and sBP (Lebaron et al., 2002; Xu et al., 2013). Vila-Costa et al. (2012) find that these HNA bacteria are phylogenetically different from LNA groups. They suggest that *Rhodobacterales*, *SAR116*, and Bacteroidetes belong to HNA bacteria, while *SAR11* and *SAR86* belong largely to LNA bacteria. Our results indicated that these active bacteria with high nucleic acid, primarily *Rhodobacteraceae* at both stations and Bacteroidetes at K11, were preferentially affected by ocean acidification, resulting in shifts in bacterial community composition and a decrease in BP. Similarly, ocean acidification is reported to inhibit the growth of a *Vibrio* sp. (Weinbauer et al., 2011), destroy the cell integrity (Coffin et al., 2004), or lower hydrolytic enzyme activities (Celussi et al., 2017). Although the high nucleic acid bacteria are preferentially grazed by predators (Gasol et al., 1999), ocean acidification did not improve protistan grazing rates in this study (Figures 5C,D). Hence, lower BP in the high-*p*CO_2_ treatment was not attributed to protistan grazing, more likely owing to the destruction of bacterial cells and inhibition of bacteria with high nucleic acid.

BR and CR responded to OA differently at both stations. OA promoted BR and CR on Day 3 at M8 but restrained BR and CR at K11 (Figure 4). At M8, the enhanced BR in the HC treatment was responsible for the slightly lower BDOC concentration in the HC treatment than in the LC treatment (Figure 2C), as BR was the major component of BCD. In contrast, at K11, OA repressed the growth of HNA bacteria, decreasing BP and BR. As a result, DOC consumption decreased in the HC treatment and BDOC in the HC treatment on Day 4 was slighter higher than in the LC treatment (Figure 2C). In turn, higher BDOC in the HC treatment mitigated sBR, as bacteria utilized BDOC at a lower energetic cost than refractory DOC (del Giorgio and Cole, 2000; Carlson et al., 2007; Xu et al., 2014). The response of bacterial respiration to OA is complicated, as the physiological response of bacterial cells to OA causes changes in BR (Bunse et al., 2016) and the quality and quantity of DOC regulate BR (Carlson et al., 2007; Xu et al., 2013). For example, OA enhances bacterial removal of DOC due to accelerated BR, which is possibly related to the enhancement of extracellular enzyme activity (James et al., 2017). Moreover, OA induces the upregulation of respiratory proton pump in bacterial transcriptome due to more H^+^, leading to an increase in BR (Bunse et al., 2016). In contrast, Teira et al. (2012) and Huang et al. (2018) report that BR decreases under high *p*CO_2_. Since OA reduces the pH of seawater, the pH is consequently the same as the intracellular pH (7.4–7.8) of bacteria, which mitigates the energetic cost to transfer H^+^ (Teira et al., 2012). Siu et al. (2014) indicate that the elevated *p*CO_2_ (1,050 ppm) induces shifts in bacterioplankton community composition, accompanied by increasing BR. Bacterial community composition differed at M8 and K11, possibly resulting in a different response of bacterial respiration to OA. Further studies were needed on the physiological response of different bacterial species to OA, which was likely important to interpret different response of bacterial respiration to OA.

Protist grazing rates decreased at M8 and increased at K11 from beginning to the end of incubation (Figures 5C,D). Elevated grazing rates of protists resulted in low BA and were partly responsible for high sBP at the end of the incubation at K11, which was 10-fold higher than that at M8 (Figures 3G,H). A reduction in BR% and an increase in CR from the beginning to the end of the incubation at K11 were linked to the increased grazing rate of protists (Figures 4, 5). BCD followed the pattern of BR, since BR was a major contributor of BCD (Figures 4I,J). However, the OA-induced decrement of BGE at both stations indicated that a greater fraction of DOC was transferred to CO_2_ in oligotrophic oceans in the OA scenario, rather than higher trophic level.

### Effect of Ocean Acidification on Bacterial Community Composition

Bacterial community compositions *in-situ* differed between M8 and K11 at the family level. *SAR11*, the most ubiquitous bacteria in the ocean (Carlson et al., 2009) dominated at both stations and followed by *Rhodospirillaceae* and *Rhodobacteraceae* at M8, both of which prefer to inhabit in nutrient-rich environments (Azam and Malfatti, 2007), and *SAR86* and *SAR116* at K11, which are oligotroph taxa (Oh et al., 2010; Giovannoni and Vergin, 2012).

The artificial operation of mesocosm resulted in conspicuous shifts in the bacterial community (Figure 7). The relative abundance of *SAR11* and *SAR116* reduced at both stations, in agreement with the observation in Sargasso Sea (Nelson and Carlson, 2012). These species inhabiting in oligotrophic oceans are less competitive in substrate-rich conditions, due to the lack of transcriptome regulatory genes (Giovannoni et al., 2005; Yooseph et al., 2010). In contrast, the relative abundance of *Rhodobacteraceae*, *Hyphomonadaceae*, and *Alteromonadaceae*, which are mainly documented in coastal or eutrophic waters (Alderkamp et al., 2006; Alonso-Gutierrez et al., 2009; Abraham and Rohde, 2014), increased in mesocosm experiments during the incubation. These opportunists are rare in natural environments, characterized by short generation times, high growth rate, and “*r*” strategy mode under artificial manipulation (Lauro et al., 2009; Hunt et al., 2010). In addition, the high relative abundance of Proteobacteria and Bacteroidetes (more than 93%) after the incubation at K11 partly resulted from the decay of phytoplankton, as indicated by the reduced Chl *a* concentration (Figure 2A). Similarly, Proteobacteria and Bacteroidetes increase in the abundance during the decay of algal blooms (Pinhassi et al., 2004).

In this study, the response of bacterial community composition to ocean acidification varied between two stations. Ocean acidification had little effect on the species diversity of the bacterial community at M8 (Figure 6A). Bacterial species responded to OA differently. The relative abundance of *Rhodobacteraceae* and *Rhodospirillaceae* decreased in HC, which might contribute to the reduced BP on Day 3, since these taxa appear to actively consume organic compounds (Alonso-Saez and Gasol, 2007). The relative abundance of *Alteromonadaceae* and *SAR86* increased under OA. *Alteromonadaceae* with relatively large cell size and the ability to thrive in nutrient-deficient situations (Lopez-Perez and Rodriguez-Valera, 2014) is repressed by ocean acidification (Xia et al., 2019). The different response of *Alteromonadaceae* to OA might result from the different environmental conditions, as Xia’s experiment is conducted during a heterotrophic bloom phase, in which BP is 20–40 folds higher than at M8. At M8, *Flavobacteriaceae* positively responded to OA on Day 2, but negatively on Day 3, which was possibly related to variability in substrates. Sala et al. (2016) find a shift in plankton community toward small phytoplankton (pico- and nanoeukaryotes) under OA. The lower abundance of large picoeukaryotes under OA is also reported by Hopkins et al. (2010). Small bacterial cells are more competitive in uptaking resource in pristine environments (Thingstad, 2003), the large-sized *Flavobacteriaceae* was less competitive than small-sized species for substrates and possibly replaced by small-sized organisms during the incubation period.

At K11, bacterial diversity decreased more rapidly in LC than in HC as BP increased more dramatically in LC during the incubation, suggesting that the significantly higher bacterial diversity in HC at K11 (Figure 6B) was more likely related to OA-induced repression of active bacteria with high nucleic acid. The higher BP and lower bacterial diversity in LC at K11 were attributed to a rapid increase in active bacteria with high nucleic acid, principally *Rhodobacteraceae*, *Alteromonadaceae*, and Bacteroidetes (Figures 3, 7). In HC, the repression of these fast-growing bacteria with high nucleic acid, which were winner of competing for substrates, favored the growth of other bacterial groups, resulting in the relatively high bacterial diversity. Enhanced bacterial diversity caused by OA is also documented (Lidbury et al., 2012). An increase in the bacterial diversity improves the resilience of the bacterial community to environmental disturbances, so-called insurance effect (Boles et al., 2004). High species richness and diversity of the bacterial community are a strategy for bacteria to resist high *p*CO_2_ and low pH (Joint et al., 2011). At K11, the relative abundance of *Rhodobacteraceae* and *Alteromonadaceae* declined under high *p*CO_2_. However, the abundance of *SAR11*, *SAR116*, and *SAR86* increased in response to OA. The abundance of these groups was correlated with bacterial metabolic parameters (BP, BR, CR, BCD, and BGE) (Figure 8B), implying that OA favored the growth of these slow-growing oligotrophic bacteria by inhibiting bacterial metabolic activities. These taxa are able to utilize a low-molecular-weight DOC that is less bioavailable (Cottrell and Kirchman, 2000; Giovannoni et al., 2005; Dupont et al., 2012). The higher abundance of “SAR” clades with small size cell and lower abundance of big-sized *Rhodobacteraceae* and *Alteromonadaceae* in HC agreed with a decrease in the relative cell size determined by flow cytometry (Figure 3J). *Hyphomonadaceae* degrade amino acids and sugars and survive across marine environments (Abraham and Rohde, 2014). A significant correlation between the abundance of *Hyphomonadaceae* and bacterial metabolic activity (BP, BCD, and BGE) implied that *Hyphomonadaceae* was linked to the decrease in bacterial metabolic activity.

### Evaluation of the Experimental Approach

The possibility that there were limitations for the short-term incubation could not be ruled out. Containment ceases the advective flow and diffusion of natural plankton (Monier et al., 2014). In our study, the short-term incubation (3–4 days) was conducted in order to mitigate the effect of the depletion of nutrient and DOC on bacterial metabolism and community composition. There also was limitation for the long-term incubation. Since nutrient and DOC concentrations play an important role in regulating bacterial metabolism, the long-term incubation exhausted substrates of natural seawater, which likely remarkably influenced bacterial activity and community. New techniques need to be developed to enable simulation experiments without changing the *in-situ* environment.

## Conclusion

Ocean acidification decreased BP and sBP, possibly since bacteria with high nucleic acid, primarily *Rhodobacteraceae*, was preferentially depressed by OA. Shifts in bacterial community composition occurred in response to OA, with higher diversity of the bacterial community in the high-*p*CO_2_ scenario. Response of BR to OA was variable, most likely associated with physiological response of different bacterial species to OA. OA reduced bacterial growth efficiency. Consequently, a greater fraction of DOC was transferred to CO_2_ in oligotrophic oceans in the OA scenario, rather than higher trophic levels.

## Data Availability Statement

The raw data supporting the conclusions of this article will be made available by the authors, without undue reservation.

## Author Contributions

CH, XL, MH, and PJ performed the bioassay experiments and measured samples. CH and JX contributed to the data analysis and manuscript writing. AL offered some valuable comments. All authors contributed to the article and approved the submitted version.

## Conflict of Interest

The authors declare that the research was conducted in the absence of any commercial or financial relationships that could be construed as a potential conflict of interest. The reviewer YZ declared a shared affiliation with one of the authors PJ to the handling editor at the time of review.

## Footnotes

1 https://github.com/OpenGene/fastp

## Supplementary Material

Supplementary Figure 1 pH **(A)** and *p*CO_2_ **(B)** during the incubation at stations M8 and K11. LC: control treatments. HC: high-*p*CO_2_ treatments. The error bars represented ± SD. For samples of HC at K11 on Day 4, *n* = 2. For other treatments, *n* = 3.

Supplementary Figure 2 Type II regression of CR_*O2*_ and CR_*INT–F*_. CR_*O2*_ derived from Winkler method and CR_*INT–F*_ calculated from ETS rates had a strong liner relationship following the equation: CR_*O2*_ = 12.4 CR_*INT–F*_ + 0.434 (*p* < 0.01, *R*^2^ = 0.88, *n* = 103). The ratio of R / ETS = 12.4.

Supplementary Figure 3 The rarefaction curve of samples during the incubation at stations M8 and K11. For samples of HC at K11 on Day 4, *n* = 2. For other treatments, *n* = 3.

Supplementary Figure 4 Bacterial species richness at M8 **(A)** and at K11 **(B)** in mesocosm experiments. LC: control treatments. HC: high-*p*CO_2_ treatments. Asterisk denoted the significant (*p* < 0.05) difference between LC and HC. The error bars represented ± SD. For samples of HC at K11 on Day 4, *n* = 2. For other treatments, *n* = 3.
