# Supplementary figures and images for: Effect of Ocean Acidification on Bacterial Metabolic Activity and Community Composition in Oligotrophic Oceans, Inferred From Short-Term Bioassays
Source: Front Microbiol. 2021 Feb 26;12:583982. doi: 10.3389/fmicb.2021.583982 (PMC7952631; doi:10.3389/fmicb.2021.583982)

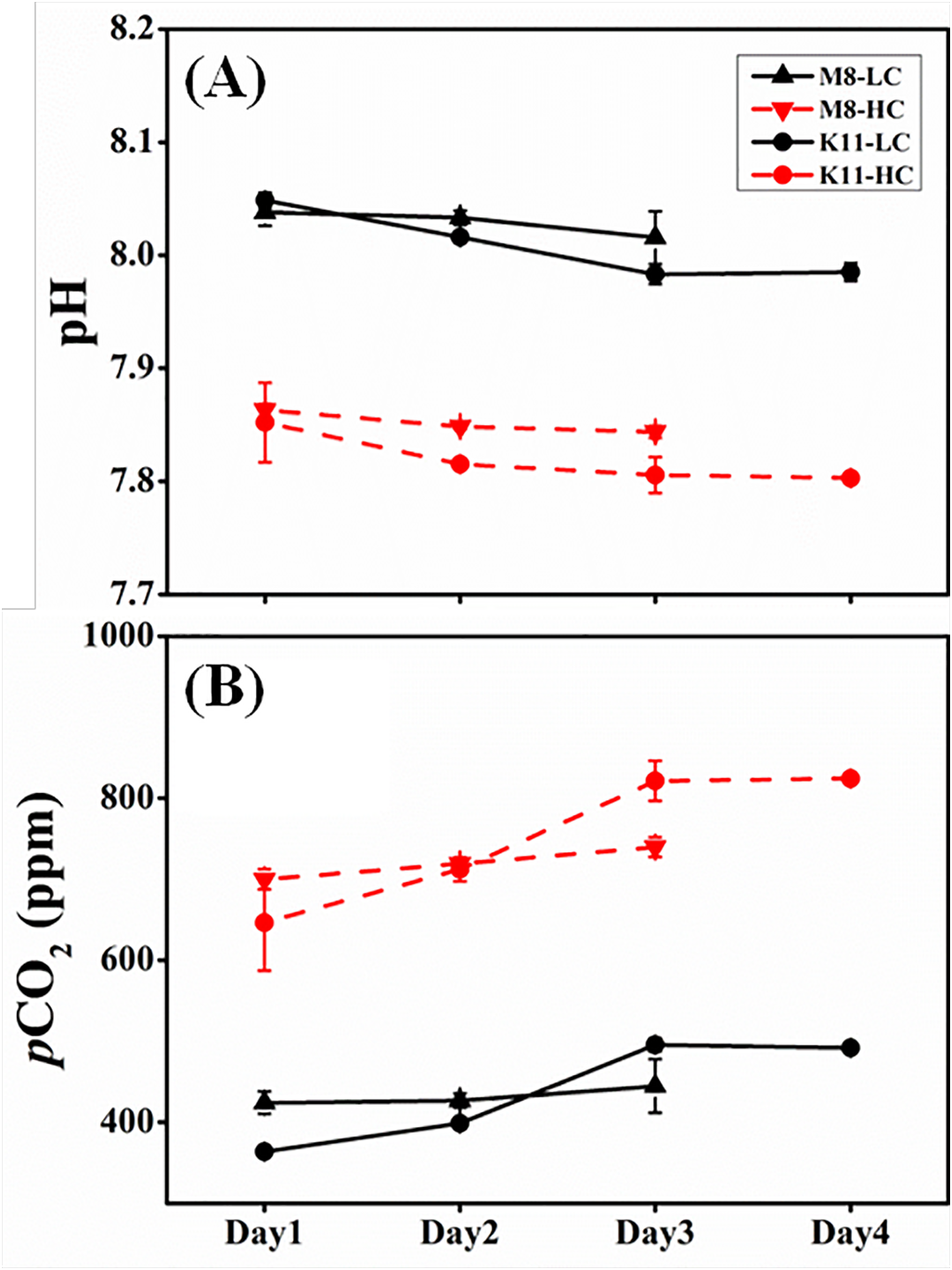

Supplement: Supplementary Figure 1 — pH (A) and pCO2 (B) during the incubation at stations M8 and K11. LC: control treatments. HC: high-pCO2 treatments. The error bars represented ± SD. For samples of HC at K11 on Day 4, n = 2. For other treatments, n = 3. [file Image_1.TIF]

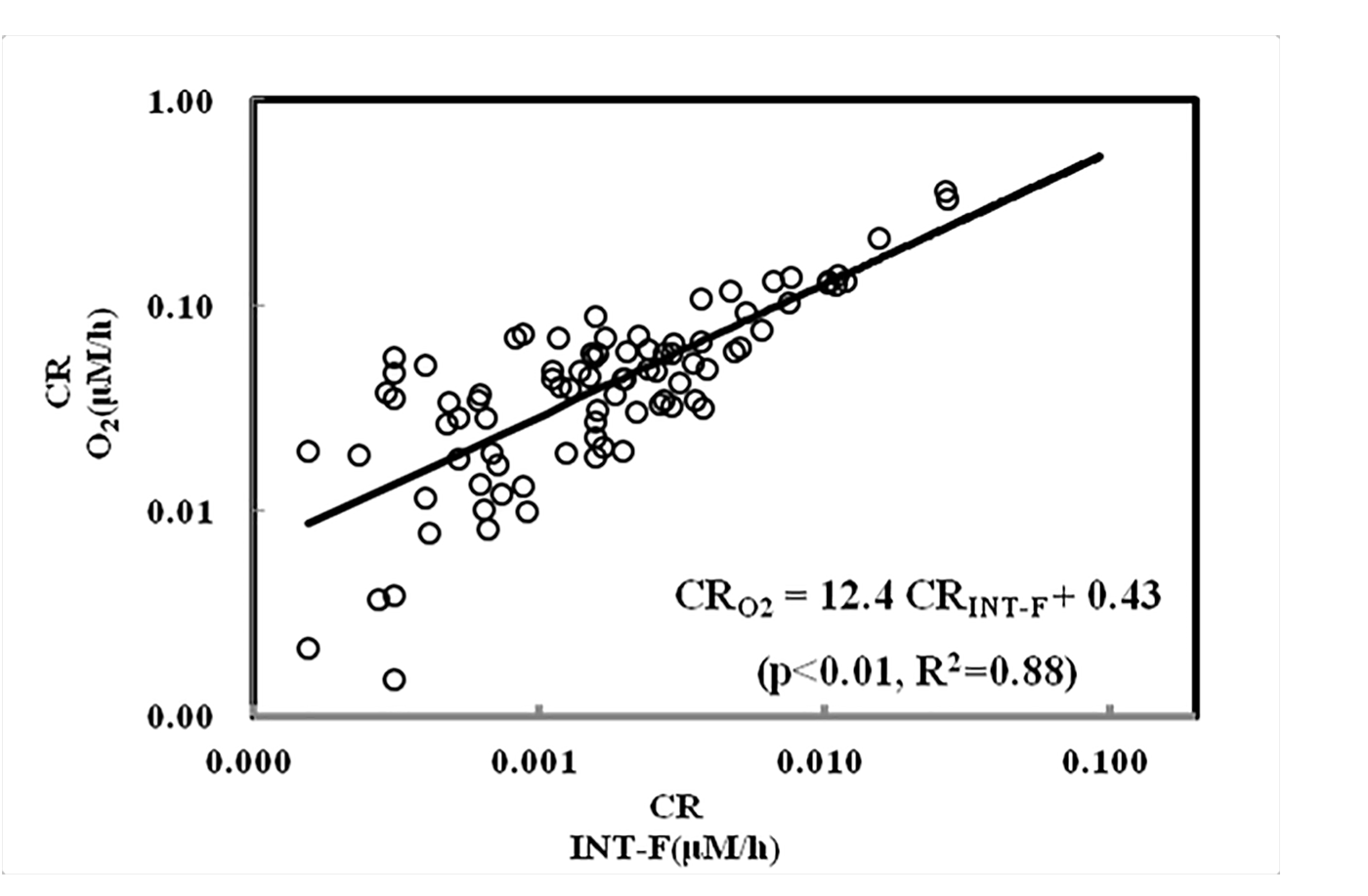

Supplement: Supplementary Figure 2 — Type II regression of CRO2 and CRINT–F. CRO2 derived from Winkler method and CRINT–F calculated from ETS rates had a strong liner relationship following the equation: CRO2 = 12.4 CRINT–F + 0.434 (p < 0.01, R2 = 0.88, n = 103). The ratio of R / ETS = 12.4. [file Image_2.TIF]

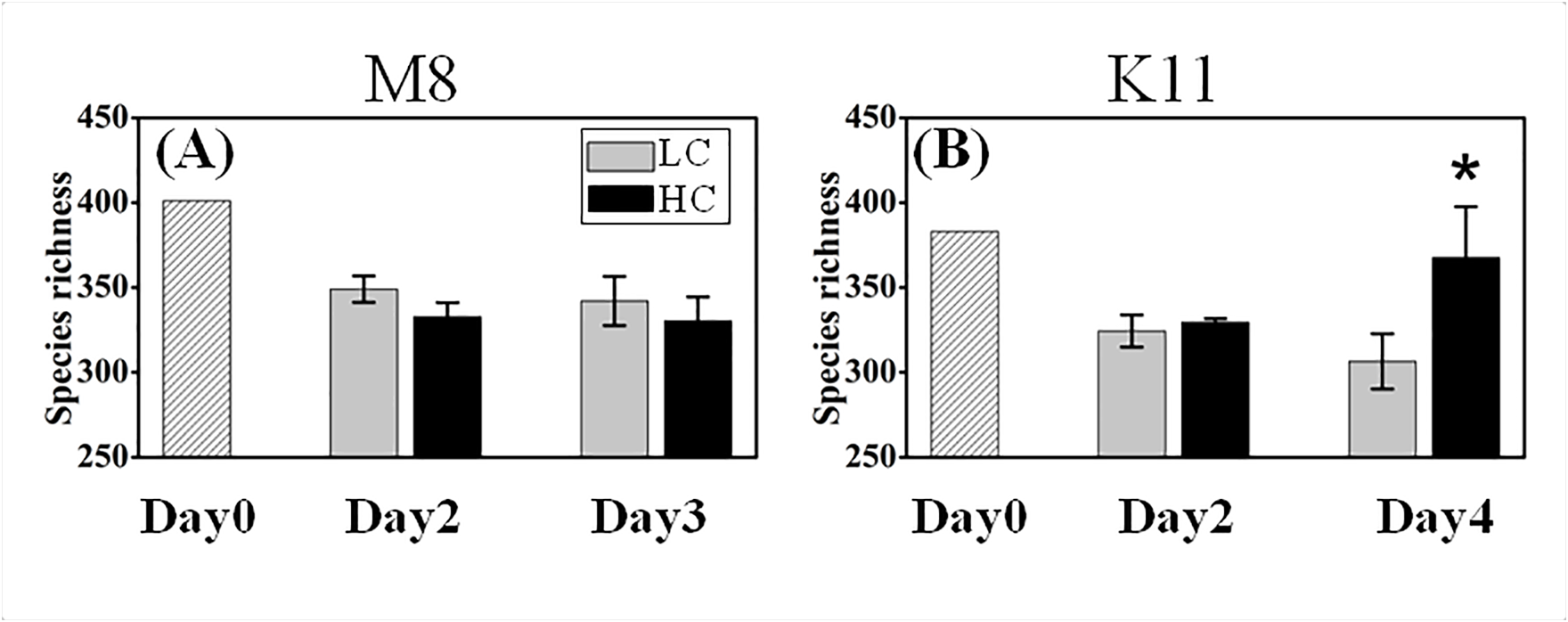

Supplement: Supplementary Figure 4 — Bacterial species richness at M8 (A) and at K11 (B) in mesocosm experiments. LC: control treatments. HC: high-pCO2 treatments. Asterisk denoted the significant (p < 0.05) difference between LC and HC. The error bars represented ± SD. For samples of HC at K11 on Day 4, n = 2. For other treatments, n = 3. [file Image_4.TIF]
